# Orthorexia nervosa and self-attitudinal aspects of body image in female and male university students

**DOI:** 10.1186/s40337-015-0038-2

**Published:** 2015-02-24

**Authors:** Anna Brytek-Matera, Lorenzo Maria Donini, Magdalena Krupa, Eleonora Poggiogalle, Phillipa Hay

**Affiliations:** Campus in Katowice, University of Social Sciences and Humanities, Katowice, Poland; Experimental Medicine Department, Sapienza University of Rome, Rome, Italy; Polish National Center for Eating Disorders, Wroclaw, Poland; Centre for Health Research, School of Medicine, University of Western Sydney and School of Medicine James Cook University, Townsville, Australia

**Keywords:** Orthorexia nervosa, Body image, Healthy food

## Abstract

**Background:**

The present study was designed to investigate orthorexia nervosa, or the phenomenon of being preoccupied with consuming healthy food. Specific aims were to explore relationships between orthorexia features and attitudes towards body image, fitness and health in normal weight female and male university students with high levels of healthy food preoccupation, i.e. orthorexia nervosa.

**Methods:**

Participants were 327 female (N = 283) and male (N = 44) students aged 18 to 25 years. All participants completed the Polish adaptation of the 15-item questionnaire assessing orthorexia eating behaviours (the ORTHO-15) and the Multidimensional Body-Self Relations Questionnaire (the MBSRQ). Relationships between scores on the ORTHO-15 and MBSRQ were explored in the 213 students who had high levels of preoccupation with a healthy food intake (68.55% women and 43.18% men, respectively).

**Results:**

There were no statistically significant differences in the levels of orthorexia behaviours between females and males. In female students with orthorexia nervosa, preoccupation with consuming healthy food was significantly correlated with the MBSRQ subscale scores for overweight preoccupation, appearance orientation, fitness orientation, health orientation, body areas satisfaction and appearance evaluation. Conversely, in male students with orthorexia nervosa there were no correlations between orthorexic behaviours and the MBSRQ subscales.

In female students with orthorexia nervosa multivariable linear regression analysis found high body areas (parts) satisfaction, low fitness orientation, low overweight preoccupation and low appearance orientation were independent predictors of greater fixation on eating healthy food. In male students, we found that aspects of body image were not associated with preoccupation with healthy eating.

**Conclusion:**

A strong preoccupation with healthy and proper food was not associated with an unhealthy body-self relationship among Polish female student with orthorexia nervosa.

## Background

Orthorexia nervosa is characterized by a “fixation on eating healthy food” and an obsession for “proper” nutrition [1]. According to Bratman and Knight [1], this eating style can be considered a psychological disorder because of physical (e.g. strict regimen of diet), psychological and social consequences (e.g. social isolation). The authors [1] proposed the following criteria for orthorexia nervosa: (a) spending an excessive amount of time (more than 3 hours per day) on thinking about looking for and preparing healthy food; (b) feeling superior to those with different eating habits; (c) following a particular health-food diet rigidly and engaging in compensatory restriction to make up for any dietary indiscretions; (d) associating self-esteem with adherence to the diet (feeling guilt when straying and self-satisfaction when complying), and (e) turning eating “properly” into the central focus of life, at the expense of other personal values, relationships, previously enjoyed activities, and sometimes physical health. Thus people with orthorexia tendencies may not be as concerned with overweight as they are self-maintaining an acceptable or low weight and they have little time or interest in other health pursuits. For Gleaves, Graham and Ambwani [2] criteria described by Bratman and Knight [1] “appear to be largely derived from the experiences of those authors; they have not been identified empirically and it has not been established that they represent a co-occurring pattern of behaviors (i. e. a syndrome)” (page 2).

**Figure 1:**
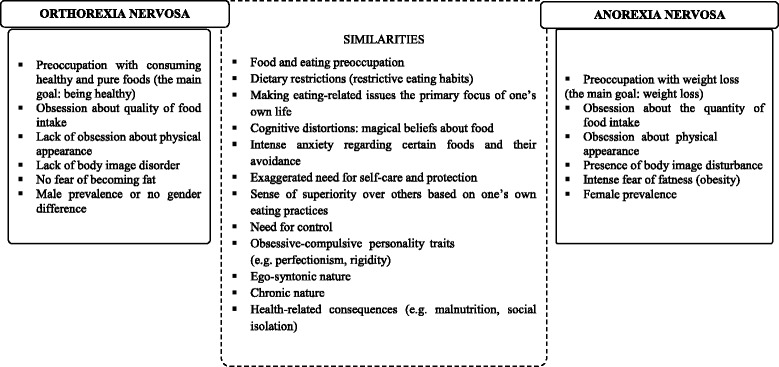
**Differences and similarities between orthorexia nervosa and anorexia nervosa [** 3 **].**

There is limited information on the categorization of orthorexia nervosa among mental disorders. An unanswered question is should be orthorexia nervosa considered as an eating disorder (at all), a variant of a currently recognized eating disorder or a separate disorder [3]? Orthorexia nervosa and anorexia nervosa share many similarities but orthorexia nervosa also differs from anorexia (Figure 1). Orthorexia might also be considered to be a particular form of “healthism” because there is an overlap between these two phenomena. Healthism is characterized by (a) health-aware and enthusiastic in seeking information about health and illness via books, magazines, Internet; (b) consumes food supplements and alternative medicines all of which are attributed ‘natural’ and ‘holistic’ qualities, and also frequently ‘detoxes’ by diet, food supplementation or other methods; and (c) makes positive lifestyle choices, e.g. takes regular exercise, has a diet that aligns with official recommendations [4]. These characteristics are also found in people with a preoccupation with consuming healthy food. The dominant research about orthorexia nervosa has taken place in Europe. Donini et al. [5] developed the ORTO-15 test based on a brief 10-item Bratman’s Orthorexia Test. A total score below 40 points implies presence of an obsessive pathological behaviour characterized by a strong preoccupation with healthy eating. The score however does not imply having a mental health disorder. To our knowledge, only four studies have been performed using the ORTO-15 test to evaluate the prevalence of a preoccupation with healthy food. A Turkish study [6] found that 43.6% of medical students showed a preoccupation with healthy food. A large Hungarian study [7] of 810 university students (89.4% female) reported over 70% having orthorexic tendencies, and American studies have reported a prevalence of orthorexic behaviours ranging from 69% [8] to 82.8% [9] among undergraduate students.

To date there is a paucity of further research on orthorexia nervosa. In contrast to the predominance of eating disorders such as anorexia nervosa in females [10, 11] findings with regard to the sex-related prevalence of preoccupation with healthy eating are inconsistent: some studies finding that males were more likely to have a higher score on the ORTO-15 test than females [5,6,12], while other papers reported that the criteria for strong preoccupation with healthy eating were met predominantly by female individuals [13,14] or no gender differences [7,9]. There might be gender differences in healthy eating resulting from food selection, food preferences and eating styles. There is a relationship between gender and specific food intake. In general, men are more likely to report eating meat (especially red meat) and hearty portion sizes and women are more likely to report consuming fruits, vegetables, fish and dairy products and smaller portion sizes [15]. In addition, women generally show a tendency to have healthier food choices than men as well as a greater awareness about diet and health-diet relationship implications [15].

Even less is known regarding the potential associations of a healthy eating preoccupation with features of other feeding and eating disorders, such as the body image concerns found in anorexia nervosa and bulimia nervosa. To our knowledge, just one study [9] has demonstrated a correlation between orthorexia nervosa and disordered eating patterns (including restraint, eating concern, weight concern, and shape concern). A study of 163 mostly female American undergraduate students [9] found no relationships between demographic features and general psychiatric symptoms and orthorexic symptoms, however higher levels of orthorexic symptoms were associated with lower levels of eating disorder symptomatology. In addition, no significant relationship was found between orthorexic symptoms and weight loss attempts [9].

Empirical studies focused on orthorexia nervosa are still very limited. In the literature links between gender and preoccupation with healthy eating are not clear and furthermore there are no studies to provide data on prevalence of orthorexia nervosa in present day Poland Therefore, our first objective was to assess and compare the rate of fixation on eating healthy food across men and women in a Polish university student population.

To our knowledge, only one empirical study [16] has shown the correlation between orthorexia nervosa features and eating and body image disturbance (i.e., if orthorexia features are present, the eating and body image disturbances are more intensive). No empirical research has investigated the relationship between orthorexia nervosa and body image, hence, the second purpose our study which was to compare levels of self-attitudinal aspects of body image in normal weight people with orthorexia nervosa.

Furthermore, to our knowledge, at present, there is only one American study [9] investigating the main features of orthorexia nervosa and their relationships with other eating and lifestyle features. These results showed that a higher level of disordered eating patterns was related to fewer orthorexia features in a sample of undergraduate students. Therefore, our third objective was to explore attitudes towards body image, fitness and health in normal weight men and women with a strong preoccupation with healthy eating.

## Methods

### Subjects

University students of Human Sciences (Psychology and Pedagogy) and Nutrition Sciences (Dietetics) from the Silesia, Lower Silesia, Mazovia and Lublin Provinces in Poland were invited to participate in the present study after being provided with written and oral information about our project. In the first phase, 396 participants were randomly selected from the four Polish universities sampling frame and of these 51 female students with eating disorders and 18 female and male students with BMI > 25 kg/m^2^ were excluded. Eating disorders were assessed on the basis of the answers to questions concerning the individual’s disordered eating pattern and negative attitudes towards body image (particularly concerning body dissatisfaction). Participants with a BMI greater than 25 kg/m^2^ (in the overweight range according to the World Health Organization’s classification [17]) were excluded in accordance with the aim to investigate orthorexia nervosa in normal weight people.

A total of 327 university students were thus recruited: 283 females and 44 males aged 18–25 years old. Oral and written informed consent was obtained from all the participants. Approval for this study was obtained from the University of Social Sciences and Humanities Human Research Ethics Committee.

The demographic characteristics of female and male students are summarized in Table 1.

**Table 1:** **Demographic and clinical features of the study sample (** ***N*** **= 327)**

**Feature**	**Women (** ***n*** **= 283)**	**Men (** ***n*** **= 44)**
Mean (*SD*)		
Age (years)	21.98 (1.74)	22.27 (2.22)
Body mass index (kg/m^2^)	21.37 (3.39)***	23.27 (4.72)
Height (m)	1.66 (0.05)***	1.80 (0.08)
Current weight (kg)	59.10 (10.23)***	75.49 (12.80)
Ideal weight (kg)	54.04 (7.81)***	75.17 (10.75)
N (%)		
Marital status		
Never married	255 (90.1%)	41 (93.2%)
Married/living as married	28 (9.9%)	2 (4.5%)
Divorced/separated/widowed	0	1 (2.3%)
Parental situation		
Parents living together	221 (78.1%)	30 (68.2%)
Divorced/separated parents	42 (14.9%)	10 (22.8%)
Deceased father	19 (6.7%)	2 (4.5%)
Deceased mother	1 (0.4%)	2 (4.5%)
Daily weighing	32 (11.3%)	4 (9.1%)
Body satisfaction present	105 (37.1%)	27 (61.4%)
Intentional weight loss with:		
Dieting	90 (31.8%)	5 (11.4%)
Physical exercise	119 (42%)	7 (15.9%)
Use of laxatives	6 (2.1%)	0
Vomiting	3 (1.1%)	0
Starvation	12 (4.2%)	0
Currently drinking alcohol	233 (82.3%)	37 (84.1%)
Currently smoking	48 (17%)	9 (20.5%)

Note: ***p < 0.001.

#### The Polish version of the ORTHO-15 test

The original ORTHO-15 test [12] is a questionnaire developed for the diagnosis of orthorexia nervosa, defined by the authors as an intense obsession for healthy food [12]. It is composed of 15 items with a four-point Likert scale with categories “always”, “often”, “sometimes”, and “never”. Items investigate the obsessive attitude of the individuals in choosing, buying, preparing and consuming food they consider to be healthy (e.g. “When you go in a food shop do you feel confused?”, “Are you willing to spend more money to have healthier food?”, “Do you think your mood affects your eating behaviour?”). A score equal to 1 corresponds to an orthorexic tendency in the eating behaviour, while a score equal to 4 points indicates normal eating habits. The authors [5] identified the threshold value below which the diagnosis of orthorexia could be given. A cut-off of 40 points indicates symptoms consistent with orthorexia nervosa [5].

In a recent study we examined the factor structure of the Polish version of the ORTHO-15 test using the exploratory factor analysis (EFA) and the confirmatory factor analysis (CFA) on two split-half study groups (N = 200 in each group) randomly selected from the larger series of 400 females and males [14]. We found that only nine items (3, 4, 5, 6, 7, 10, 11, 12 and 14) out of 15, determined the structure of the Polish version of the ORTHO-15 test [14]. Cronbach’s alpha coefficient for the Polish version of the ORTHO-15 was .644 [14]. Adapting the Donini et al. [4] cutoff of a score of 40 out of a maximum score of 60 we calculated that in our Polish population a score of < 24 indicated a strong preoccupation with consuming healthy food (in our study the maximum score was 36).

#### The Multidimensional Body-Self Relations Questionnaire

The Multidimensional Body-Self Relations Questionnaire (MBSRQ) [18] is a 69-item self-report inventory for evaluating attitudes related to body image. The MBSRQ consists of 10 subscales.

The Appearance evaluation subscale measures feelings of physical (un)attractiveness, (dis)satisfaction with one’s looks (e.g. “Most people would consider me good-looking”). High scorers feel most of the time positive and satisfied with their appearance. The Appearance orientation subscale assesses the extent of investment in one’s appearance (e.g. “I check my appearance in a mirror whenever I can”). High scorers place more importance on how they look and engage in extensive grooming behaviour.

The Fitness evaluation subscale evaluates feelings of being physically (un)fit (e.g. “I do poorly in physical sports or games”). High scorers regard themselves as physically fit regularly incorporate exercise activities into their lifestyle. The Fitness orientation subscale measures the extent of investment in being physically fit or athletically competent (e.g. “I try to be physically active”). High scorers value fitness and are actively involved in activities to enhance or maintain their fitness.

The Health evaluation subscale which assesses feelings of physical health and/or the freedom from physical illness (e.g. “I am seldom physically ill”). High scorers feel their bodies are in good health. The Health orientation subscale reflects the extent of investment in a physically healthy lifestyle (e.g. “I know a lot about physical fitness”). High scorers are try to lead a healthy lifestyle. The illness orientation subscale which evaluates extent of reactivity to being or becoming ill (e.g. “I pay close attention to my body for any signs of illness”). High scorers are apt to seek medical attention.

The Body areas satisfaction subscale assesses satisfaction with discrete parts of one’s appearance (e.g. face, upper torso). High composite scorers are generally content with most areas of their body.

The Overweight preoccupation subscale measures a construct reflecting fat anxiety, weight vigilance, dieting, and eating restraint (e.g. “I constantly worry about being or becoming fat”). The Self-classified weight subscale reflects how one perceives and labels one’s weight, from very underweight to very overweight. (e.g. “From looking at me, most other people would think I am …”) [18].

Internal consistency alpha for the subscales of the MBSRQ ranged from .70 to .91 for males and from .73 to .90 for females [14]. The MBSRQ was used in this study with permission from the author. The Polish version of the MBSRQ was translated by Schier [19].

### Statistics

Data were entered into a database and analyzed using the Statistical Package for the Social Sciences (SPSS version 19.0 for Windows). Data were inspected for normality. The results for each study group were compared using the *t*-test for unpaired data. The relationships between different variables were evaluated by Pearson’s correlation coefficient (*r*_*p*_). Multiple regression analysis (stepwise multiple regression) was performed to identify determinants of orthorexia nervosa in female and male students. A significance level (*p*) of 0.05 was assumed.

## Results

### Participant selection

**Figure 2:**
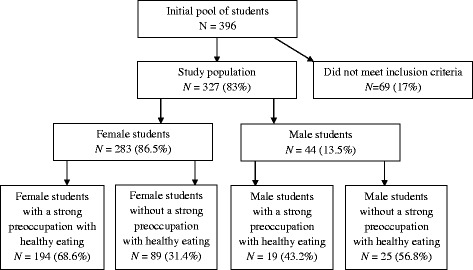
**Participant recruitment flow chart.**

The study sample was 327 female and male students aged from 18 to 25 years. The mean age was 21.98 years (*SD* = 1.74) and 22.27 years (*SD* = 2.22) for female and male students, respectively. Two hundred and thirteen participants were preoccupied with consuming healthy food based on score below cut-off of 24 points on the Polish adaptation of the ORTO-15 test (68.6% women and 43.2% men, respectively) (Figure 2). These participants comprised the sample for the studies of normal weight people with orthorexia nervosa. The average age of the two groups presenting a strong preoccupation with healthy eating was 21.98 years (*SD* = 1.77) and 22.63 years (*SD* = 2.85) for female and male students, respectively.

### Comparative study

In the comparative analysis we only included female and male students showing a preoccupation with healthy food. Means and standard deviations for the Polish adaptation of the ORTO-15 test and the MBSRQ among female and male students with orthorexia nervosa are shown in Table 2.

**Table 2:** **Orthorexia questionnaire (ORTHO-15) and multidimensional body-self relations questionnaire (MBSRQ) subscale scores of the 213 students with a strong preoccupation with healthy eating**

	**Female students n = 194**	**Male students n = 19**	***t*** **(all df = 211)**	***p***
	**Mean (** ***SD*** **)**			
Total ORTO-15 score	19.66 (2.82)	19.42 (2.71)	0.360	0.719
MBSRQ subscale scores				
Appearance evaluation	3.16 (0.93)	3.77 (0.75)	−2.72	0.007
Appearance orientation	3.84 (0.56)	3.05 (0.73)	5.67	0.000
Fitness evaluation	3.25 (0.98)	4.10 (0.76)	−3.67	0.000
Fitness orientation	3.27 (0.80)	4.06 (0.65)	−4.17	0.000
Health evaluation	3.46 (0.81)	4.29 (0.53)	−4.39	0.000
Health orientation	3.13 (0.45)	3.41 (0.36)	−2.60	0.010
Illness orientation	3.17 (0.75)	2.91 (0.74)	1.44	0.150
Overweight preoccupation	2.93 (1.03)	1.80 (0.62)	4.66	0.000
Self-classified weight	3.18 (0.61)	2.78 (0.69)	2.66	0.008
Body areas satisfaction	3.16 (0.68)	3.64 (0.62)	−2.95	0.004

The total score of the Polish adaptation of the ORTO-15 test was not significantly different between female and male participants (*p* = 0.719).

The orthorexia group of female students reported significantly lower appearance evaluation scores (*p* = 0.007), fitness evaluation scores (*p* < 0.001) and health orientation scores (*p* = 0.01), than male students. They also had lower ratings on fitness orientation (*p* < 0.001), lower scores on health evaluation (*p* < 0.001) and lower ratings on body areas satisfaction (*p* = 0.004) than male students. Female students with a strong preoccupation with healthy eating reported higher scores on appearance orientation (*p* < 0.001), overweight preoccupation (*p* < 0.001), and higher self-classified weight (*p* = 0.008) in comparison with male students with a strong preoccupation with healthy eating. There was no sex difference for scores on illness orientation (*p* = 0.15).

### Correlation study

The correlations between the Polish adaptation of ORTO-15 test and the MBSRQ in students with fixation on eating healthy food are presented in Table 3.

**Table 3:** **Pearson correlations (r**_p_ **) between othorexia nervosa symptom scores as measured on the ortho-15 and the multidimensional body-self relations questionnaire (MBSRQ) subscale scores among the 213 students with a strong preoccupation with healthy eating**

**MBSRQ subscale score**	**Female students** ***n*** **= 194**		**Male students** ***n*** **= 19**	
	***r***_p_	***p***	***r***_p_	***p***
Appearance evaluation	0.159	0.027	−0.149	0.543
Appearance orientation	−0.347	0.000	−0.252	0.297
Fitness evaluation	−0.087	0.228	−0.130	0.596
Fitness orientation	−0.332	0.000	−0.203	0.406
Health evaluation	−0.017	0.814	−0.021	0.932
Health orientation	−0.218	0.002	−0.429	0.067
Illness orientation	−0.072	0.319	−0.344	0.150
Overweight preoccupation	−0.400	0.000	−0.161	0.511
Self-classified weight	−0.078	0.279	0.005	0.982
Body areas satisfaction	0.226	0.002	−0.374	0.115

In female students a strong preoccupation with healthy eating was negatively related to overweight preoccupation, appearance orientation, fitness orientation, health orientation, body areas satisfaction and appearance evaluation. However, in male students we did not find any significant correlation between orthorexia nervosa and the scores at MBSRQ subscales.

### Regression analysis for orthorexia nervosa

Because we aimed to investigate which factors were related with orthorexia nervosa symptomatology in female and male students, the regression study used only participants within the cut-off range. The results of the stepwise regression analysis (Table 4) indicated that body areas satisfaction (*β* = 0.169), fitness orientation (*β* = −0.327), overweight preoccupation (*β* = −0.210) and appearance orientation (*β* = −0.186) were predictors of a strong preoccupation with healthy eating among female students. Results of the regression analysis were statistically significant, *F *(1,192) = 19.78, *p* < 0.001. Taken together, these variables predicted 29% of the variance (*R*^2^ = 0.295).

**Table 4:** **Stepwise regression analyses for predicting a strong preoccupation with healthy eating in female students**

**Independent variables**	**B**	**SE**	**Beta**	***R*** ^*2*^	**Adj.** ***R*** ^*2*^	***F***	***P*** **value**
Female students (n = 194)							
Step 1				0.160	0.155	36.49	0.000
Overweight preoccupation	−1.09	0.181	−0.400				
Step 2				0.246	0.238	32.20	0.000
Overweight preoccupation	−1.01	0.173	−0.370				
Fitness orientation	−1.04	0.222	−0.296				
Step 3				0.275	0.264	24.03	0.000
Overweight preoccupation	−0.799	0.187	−0.292				
Fitness orientation	−0.984	0.219	−0.280				
Appearance orientation	−0.946	0.344	−0.188				
Step 4				0.295	0.280	19.78	0.000
Overweight preoccupation	−0.574	0.208	−0.210				
Fitness orientation	−1.14	0.228	−0.327				
Appearance orientation	−0.936	0.341	−0.186				
Body areas satisfaction	0.697	0.301	0.169				

Attitudes related to body image did not influence a strong preoccupation with healthy eating in males (*F *(10,8) = 0.860, *p* = 0.597).

## Discussion and conclusion

In this study a majority (68.55%) of female students and large minority (43.18%) of male counterparts reported met criteria for high levels of orthorexic (healthy eating) behaviours. This may have been because the study selected students who were mostly studying psychology or dietetics and thus already were knowledgeable and interested in nutrition, health and well-being. Our findings are consistent with Baĝci Bosi et al. [20], who found people with more education about healthy nutrition were more likely to be concerned with healthy and proper nutrition and to be fixated on eating healthy food. Fidan et al. [6] also found that the prevalence of fixation on eating healthy food was higher among medical male students than medical female students, and a high prevalence of orthorexic behaviours (43.6%) was observed globally among medical students. Nutrition students in particular may thus be a high risk group to become preoccupied with consuming healthy food [6,21].

As in other studies [7,9] we found no differences in the levels of healthy eating preoccupation between males and females. However, caution must be applied to the findings as there were an unequal number of female and male participants, likely due to the female predominance in the disciplines of psychology and dietetics at the university. Our findings do not support Donini et al. [5,11] who reported a higher prevalence of healthy eating preoccupation in Italian men. Conversely they also differ from Arusoğlu et al. [13], who found a female predominance of healthy eating in a more representation general population sample in Turkey. Differences in gender distribution may be because of different methods of sample recruitment and selection. For example, Donini and colleagues [5,11] recruited from both students and employees of diverse institutions and parents of students at a high school.

In the present study we found that women with orthorexia nervosa were less likely to: (1) regularly incorporate exercise activities into their lifestyle (fitness orientation subscale), (2) concentrate on dieting, eating restraint and weight vigilance (overweight preoccupation subscale), (3) pay attention to their appearance (appearance orientation subscale) and (4) lead a physically healthy lifestyle (health orientation subscale). At first sight, these results seem to be counterintuitive. They question (along with its high prevalence) the nature of orthorexia nervosa and that it may be a particular health preoccupation that takes precedence over other aspects of health. At this stage interpretations can only however be speculative and more research is needed.

Despite the fact that orthorexia nervosa is characterized by a strong preoccupation with healthy eating, the literature is not clear about whether this phenomenon is an eating disorder at all, a variant of a currently recognized eating disorder, a variant of obsessive-compulsive disorder, a separate disorder or a disorder precursor. Our results indicated that in female students, a strong preoccupation with healthy eating was positively correlated with appearance evaluation and body areas satisfaction. This relationship could indicate that as female students’ satisfaction with the size and physical attractiveness increases, their preoccupation with healthy food increases. These findings may be explained by the fact that similar to people with anorexia nervosa, people with orthorexia nervosa experience their symptoms about body image as ego-syntonic (relating to aspects of a people’s thoughts, behaviour, attitudes, feelings viewed as acceptable and consistent with the self-conception) [2].

MacEvilly [22] suggested that orthorexia nervosa should be considered as a risk factor or early phase in the development of an eating disorder rather than classifying it as an eating disorder. Moreover, it does not include the most characteristic symptoms of anorexia nervosa and bulimia nervosa (i.e., preoccupation with weight loss, immense fear of becoming fat, and/or overestimation of body size) [23] and it does not start with low self-esteem [22]. In contrast to eating disorders which involve obsessions about the quantity of food intake, orthorexia nervosa results from an obsession about the quality of food intake [1,5]. Furthermore, (and consistent with our findings) orthorexia nervosa is related to the consumption of healthy and pure foods with the aim of being healthy (and not losing weight like in the case of eating disorders). In some respects orthorexia nervosa is more similar to the newly introduced Avoidant Restrictive Food Intake Disorder or ARFID where there is also an absence of body image concern [24]. However in ARFID the dietary restriction may often be driven by a food anxiety or fear rather than health preoccupation.

The present study was among the first to investigate the relationship between orthorexia nervosa and body image in both males and females. Limitations include: (1) we used only self-report measures; (2) we applied a small number of questionnaires; (3) the study was cross-sectional and could not assess causality of relationships; (4) females were more represented numerically than males in the university student population considered which was a sample of students was not representative of the general population; and (5) we only recruited university students of Human and Nutrition Sciences (leading to a sampling bias of psychology and dietetics students).

Further studies are needed to explore the relationship between body image and a strong preoccupation with healthy eating in different populations, including samples that include people who are overweight and/or have an eating disorder, and to investigate relationships more broadly between orthorexia tendencies and other factors such as perfectionism, self-esteem and self-control (which are frequently cited in the literature as the personality traits associated with orthorexia nervosa [23,25,26]). Research is needed to characterize the features and the determinants of orthorexia nervosa and test the hypothesis that orthorexia as a dimensional construct may be a phenomena with positive, neutral or negative impact on adaptive function and quality of life.
