# Erratum to: Orthorexia nervosa and self-attitudinal aspects of body image in female and male university students

**DOI:** 10.1186/s40337-016-0105-3

**Published:** 2016-05-16

**Authors:** Anna Brytek-Matera, Lorenzo Maria Donini, Magdalena Krupa, Eleonora Poggiogalle, Phillipa Hay

**Affiliations:** Campus in Katowice, University of Social Sciences and Humanities, Katowice, Poland; Experimental Medicine Department, Sapienza University of Rome, Rome, Italy; Polish National Center for Eating Disorders, Wroclaw, Poland; Centre for Health Research, School of Medicine, University of Western Sydney and School of Medicine James Cook University, Townsville, Australia

Erratum

Unfortunately, the original version of this article [1] contained an error. After publication it came to the author’s attention that the interpretation of the correlational analyses in this paper was incorrect because the ORTHO-15 test is reversed scored.

The Abstract results should report that:

“In female students with orthorexia nervosa multivariable linear regression analysis found low body areas (parts) satisfaction, high fitness orientation, high overweight preoccupation and high appearance orientation were independent predictors of greater fixation on eating healthy food.”

And conclusion that:

“A strong preoccupation with healthy and proper food was associated with an unhealthy body-self relationship among Polish female student with orthorexia nervosa.”

The reporting of results on page 5 is correct. Because the correlations were negative the interpretation in the discussion should be corrected to indicate that in female students, a strong preoccupation with healthy eating was associated with lower appearance evaluation and body areas satisfaction and women with orthorexia nervosa were more likely to: (1) regularly incorporate exercise activities into their lifestyle (fitness orientation subscale), (2) concentrate on dieting, eating restraint and weight vigilance (overweight preoccupation subscale), (3) pay attention to their appearance (appearance orientation subscale) and (4) lead a physically healthy lifestyle (health orientation subscale). These findings are not counterintuitive, but support the hypothesis that there is an association between orthorexia and features of disordered eating and body image concern.

## References

[1] Brytek-Matera A, Donini LM, Krupa M, Poggiogalle E, Hay P (2015). Orthorexia nervosa and self-attitudinal aspects of body image in female and male university students. J Eat Disord.

